# Azathioprine favourably influences the course of malaria

**DOI:** 10.1186/1475-2875-8-102

**Published:** 2009-05-14

**Authors:** Diwakar Bobbala, Saisudha Koka, Corinna Geiger, Michael Föller, Stephan M Huber, Florian Lang

**Affiliations:** 1 Department of Physiology, University of Tübingen, Gmelinstr. 5, D-72076, Tübingen, Germany; 2 Department of Radiation Oncology, University of Tübingen, Tübingen, Germany

## Abstract

**Background:**

Azathioprine triggers suicidal erythrocyte death or eryptosis, characterized by cell shrinkage and exposure of phosphatidylserine at the erythrocyte surface. Eryptosis may accelerate the clearance of *Plasmodium*-infected erythrocytes. The present study thus explored whether azathioprine influences eryptosis of *Plasmodium*-infected erythrocytes, development of parasitaemia and thus the course of malaria.

**Methods:**

Human erythrocytes were infected *in vitro *with *Plasmodium falciparum (P. falciparum) *(strain BinH) in the absence and presence of azathioprine (0.001 – 10 μM), parasitaemia determined utilizing Syto16, phosphatidylserine exposure estimated from annexin V-binding and cell volume from forward scatter in FACS analysis. Mice were infected with *Plasmodium berghei (P. berghei) *ANKA by injecting parasitized murine erythrocytes (1 × 10^6^) intraperitoneally. Where indicated azathioprine (5 mg/kg b.w.) was administered subcutaneously from the eighth day of infection.

**Results:**

*In vitro *infection of human erythrocytes with *P. falciparum *increased annexin V-binding and initially decreased forward scatter, effects significantly augmented by azathioprine. At higher concentrations azathioprine significantly decreased intraerythrocytic DNA/RNA content (≥ 1 μM) and *in vitro *parasitaemia (≥ 1 μM). Administration of azathioprine significantly decreased the parasitaemia of circulating erythrocytes and increased the survival of *P. berghei*-infected mice (from 0% to 77% 22 days after infection).

**Conclusion:**

Azathioprine inhibits intraerythrocytic growth of *P. falciparum*, enhances suicidal death of infected erythrocytes, decreases parasitaemia and fosters host survival during malaria.

## Background

Infection of erythrocytes with *Plasmodium falciparum *stimulates eryptosis, the suicidal death of erythrocytes [1,2]. Eryptosis is characterized by cell membrane scrambling leading to phosphatidylserine exposure at the cell surface [3-7]. Triggers of cell membrane scrambling include increased cytosolic Ca^2+ ^activity [3,5,6,8] and ceramide [9]. Ca^2+ ^may enter erythrocytes through Ca^2+^-permeable cation channels, which could be activated by osmotic shock, oxidative stress or energy depletion [8,10-12]. Ca^2+ ^further activates Ca^2+^-sensitive K^+ ^channels [13,14], leading to exit of KCl and osmotically obliged water and thus to cell shrinkage [15]. *Plasmodium *infection imposes oxidative stress onto host erythrocytes, which activates Ca^2+^-permeable cation channels [16] and, thus, fosters cell membrane scrambling and phosphatidylserine exposure at the erythrocyte surface [17]. Sustained increase in cytosolic Ca^2+ ^similarly stimulates apoptosis of nucleated cells [18]. As phosphatidylserine-exposing cells are bound to receptors of macrophages [19,20] and phagocytosed [21,22], eryptotic cells are rapidly cleared from circulating blood [23].

During malaria, the clearance of infected erythrocytes prior to the development of trophozoites [24] may counteract the development of parasitaemia [25]. Along those lines clearance of ring stage *Plasmodium*-infected erythrocytes is accelerated by sickle-cell trait, beta-thalassaemia-trait, homozygous Hb-C and G6PD-deficiency, genetic conditions associated with a relatively mild course of malaria [7,26-30]. Moreover, iron deficiency [1] and treatment with lead [2], chlorpromazine [31] and cyclosporine [32] delay the development of parasitaemia and thus foster the survival of *Plasmodium berghei*-infected mice, presumably at least in part by accelerating erythrocyte death. Erythropoietin, which inhibits the erythrocyte cation channel [33] has similarly been shown to influence the course of malaria [34]. Erythropoietin may, however, be effective through mechanisms other than stimulation of eryptosis, which is rather inhibited by the hormone [33].

Azathioprine, a widely used immunosuppressive drug [35-38], has recently been shown to similarly trigger eryptosis [39]. The present study explored whether azathioprine accelerates eryptosis of *P. falciparum*-infected erythrocytes and whether it influences parasitaemia and survival during malaria. Azathioprine (6-mercaptopurine) has previously been shown to inhibit a purine phosphoribosyltransferase of the parasite and thus to interfere with *in vitro *growth of the parasite [40,41]. An effect on the survival of infected erythrocytes or *in vivo *efficacy has, however, not been reported.

## Methods

### Animals, cells and solutions

Animal experiments were performed according to the German animal protection law and approved by the local authorities (registration number PY 2/06). Experiments were performed in healthy SV129/J wild type mice (aged 4 months, both male and female). The animals had free access to standard chow (C1310, Altromin, Lage, Germany) and drinking water. Murine erythrocytes were drawn from the animals by incision of the tail vein.

Human erythrocytes were drawn from healthy volunteers.

Experiments were performed at 37°C in Ringer solution containing (in mM) 125 NaCl, 5 KCl, 1 MgSO_4_, 32 HEPES/NaOH (pH 7.4), 5 glucose, 1 CaCl_2_. Azathioprine was added to the NaCl Ringer at final concentrations varying from 0.001 μM to 10 μM (Sigma, Schnelldorf, Germany). For *in vitro *azathioprine treatment, the final haematocrit was adjusted to 0.3%.

### Determination of phosphatidylserine exposure

FACS analysis was performed as described [8]. After incubation in the presence or absence of azathioprine, suspensions of *P. falciparum*-infected erythrocytes were stained with annexin V-APC (BD Biosciences Pharmingen, Heidelberg, Germany) and/or with the DNA/RNA specific dye Syto16 (Molecular Probes, Göttingen, Germany) to identify phosphatidylserine-exposing and infected erythrocytes, respectively. For annexin V-binding, erythrocytes were washed, resuspended in annexin V-binding buffer (Ringer solution containing 5 mM CaCl_2_. pH 7.4), stained with annexin V-APC (dilution 1:20), incubated for 20 min at room temperature, and diluted 1:5 with annexin V-binding buffer. Syto16 (final concentration of 20 nM) was added directly to the diluted erythrocyte suspension or co-incubated in the annexin V-binding buffer. Cells were analyzed by flow cytometry (FACS-Calibur, BD) in FL-1 for Syto16 (detected at 530 nm) and in FL-4 for annexin V-APC fluorescence intensity (detected at 660 nm).

### *In vitro *cultivation of *Plasmodium falciparum*

For infection of human erythrocytes, the human pathogen *P. falciparum *strain BinH [42] was grown *in vitro *[43]. Parasites were cultured as described earlier [44,45] at a haematocrit of 2% and a parasitaemia of 2–10% in RPMI 1640 medium supplemented with Albumax II (0.5%; Gibco, Karlsruhe, Germany) in an atmosphere of 90% N_2_, 5% CO_2_, 5% O_2_.

### *In vivo *proliferation of *Plasmodium berghei*

For infection of mice, *P. berghei *ANKA-parasitized murine erythrocytes (1 × 10^6^) were injected intraperitoneally [46,47] into wild-type mice. Where indicated, azathioprine (5 mg/kg b.w) was administered subcutaneously from the eighth day of infection. Blood was collected from the mice starting on the 8th day after infection. Parasitaemia was determined by Syto-16 staining in FACS analysis.

### *In vitro *growth assays of *P. falciparum*-infected human erythrocytes

The *P. falciparum *BinH strain was cultured and synchronized to the ring stage by sorbitol treatment as described previously [16]. For the *in vitro *growth assay, synchronized parasitized erythrocytes were aliquoted in 96-well plates (200 μl aliquots, 1% haematocrit, 0.5–2% parasitaemia) and grown for 48 h in the presence or absence of azathioprine (0.001 μM – 10 μM). The parasitaemia was assessed at time 0 and after 48 h of culture by flow cytometry. Parasitaemia was defined by the percentage of erythrocytes stained with the DNA/RNA specific fluorescence dye Syto16.

To estimate DNA/RNA amplification of the intraerythrocytic parasite, the culture was ring stage-synchronized, and re-synchronized after 6 h of culture (to narrow the developmental parasite stage), aliquoted (200 μl aliquots, 2% haematocrit and 10% parasitaemia) and cultured for further 16 h in the presence or absence of azathioprine (0.001 μM – 10 μM). Thereafter, the DNA/RNA amount of the parasitized erythrocytes was determined by Syto16 fluorescence as a measure of intraerythrocytic parasite copies.

### Statistics

Data are expressed as arithmetic means ± SEM and statistical analysis was made by t-test or ANOVA using Tukey's test as post hoc test, as appropriate. p < 0.05 was considered as statistically significant.

## Results

**Figure 1:**
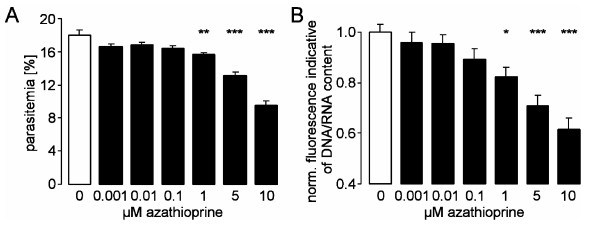
**Effects of azathioprine on intraerythrocytic amplification and *in vitro *parasitaemia**. **A. ***In vitro *parasitaemia with *P. falciparum *in human erythrocytes as a function of the azathioprine concentration (arithmetic means ± SEM, n = 8). * indicates significant difference (p ≤ 0.05) from absence of azathioprine. **B.** Intraerythrocytic DNA amplification as a function of the azathioprine concentration (arithmetic means ± SEM, n = 6).

To study the *in vitro *growth of the parasite, *P. falciparum*-infected erythrocytes were cultured in healthy human erythrocytes and synchronized to ring stage by sorbitol treatment. The initial parasitaemia was 1.3%. Within 48 hours of culture, i.e., after intraerythrocytic amplification, evasion from the host cell, and invasion into new erythrocytes, some 16% of the erythrocytes were infected, while 84% of the erythrocytes remained noninfected (Figure 1A). The percentage of parasitized erythrocytes was decreased by the presence of azathioprine, an effect reaching statistical significance at ≥ 1 μM azathioprine concentration (Figure 1A). Similarly, the intraerythrocytic DNA amplification of the parasite was decreased in the presence of azathioprine, an effect reaching statistical significance at ≥ 1 μM azathioprine concentration (Figure 1B). Together, the data indicate that azathioprine exerts direct effects on the parasite at concentrations ≥ 1 μM.

**Figure 2:**
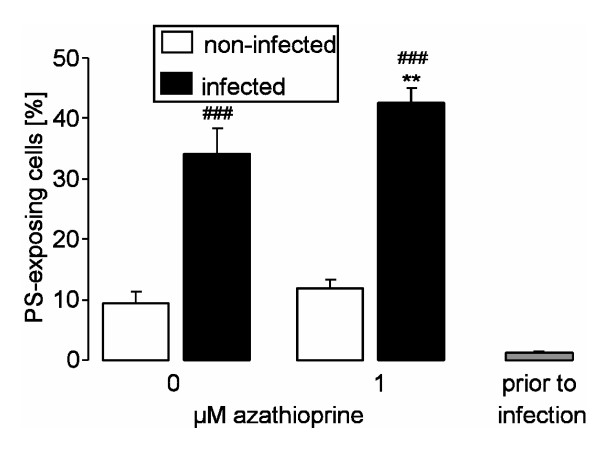
**Effects of azathioprine on phosphatidylserine exposure of infected and noninfected erythrocytes**. Arithmetic means ± SEM (n = 12) of annexin V-binding of infected (closed bars) and noninfected (open bars) erythrocytes following infection of human erythrocytes with *P. falciparum *at 0 μM (left bars) and 1 μM azathioprine. For comparison, the percentage of annexin V-binding erythrocytes prior to infection is shown (grey bar). ### indicates significant difference (p ≤ 0.001; paired ANOVA) from noninfected erythrocytes, ** indicates significant difference (p ≤ 0.01; paired ANOVA) from absence of azathioprine.

To explore whether infection of erythrocytes triggers eryptosis, phosphatidylserine-exposing erythrocytes were identified by determination of annexin V-binding in FACS analysis. Prior to infection, the percentage of annexin V-binding erythrocytes was low (1.25 ± 0.20%, n = 6). Infection within 24 hours led to a marked increase in annexin V-binding of both, infected erythrocytes and noninfected bystander cells (Figure 2). The percentage of annexin V-binding was more than double as high in infected than in noninfected erythrocytes (Figure 2), a difference statistically significant both, in the absence and presence of azathioprine. The phosphatidylserine exposure of infected erythrocytes was significantly augmented by azathioprine (Figure 2), an effect observed at 1 μM azathioprine.

**Figure 3:**
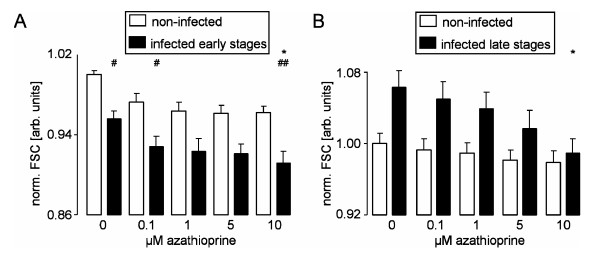
**Effects of azathioprine on forward scatter of infected and noninfected erythrocytes**. **A.** Normalized forward scatter (n = 12) of the early stage-infected erythrocytes (closed symbols) and noninfected (open symbols) erythrocytes as a function of the azathioprine concentration. * indicates significant difference (p ≤ 0.05; ANOVA) from absence of azathioprine, ^#^, ^## ^indicate significant difference (p ≤ 0.05, p ≤ 0.01; ANOVA) from noninfected erythrocytes. Noninfected erythrocytes and erythrocytes infected with early and late parasite stages were defined by background, intermediate and high staining of the cells with the DNA/RNA-specific fluorescence dye Syto16. **B.** Normalized forward scatter (n = 12) of late stage-infected erythrocytes (closed symbols) and noninfected (open symbols) erythrocytes as a function of the azathioprine concentration. * indicates significant difference (p ≤ 0.05; ANOVA) from absence of azathioprine.

Depending on the stage of the parasite development, infection of erythrocytes decreased (early stages; Figure 3A) or increased (late stages; Figure 3B) erythrocyte forward scatter, indicating that early stages initially decreased the host cell volume. Subsequently, during later parasite development, the volume-expanding trophozoites increased the host cell volume. Azathioprine at concentrations of 5 and 10 μM decreased the forward scatter of late stage infected erythrocytes, which was probably due to azathioprine-induced inhibition of intraerythrocytic parasite development (see Figure 1B). In the early stage of infection, however, a statistically significant shrinking effect of azathioprine on infected cells was evident at lower concentrations of azathioprine (≥ 0.1 μM). In summary, these experiments indicate that low concentrations of azathioprine augment eryptosis of the host erythrocyte.

**Figure 4:**
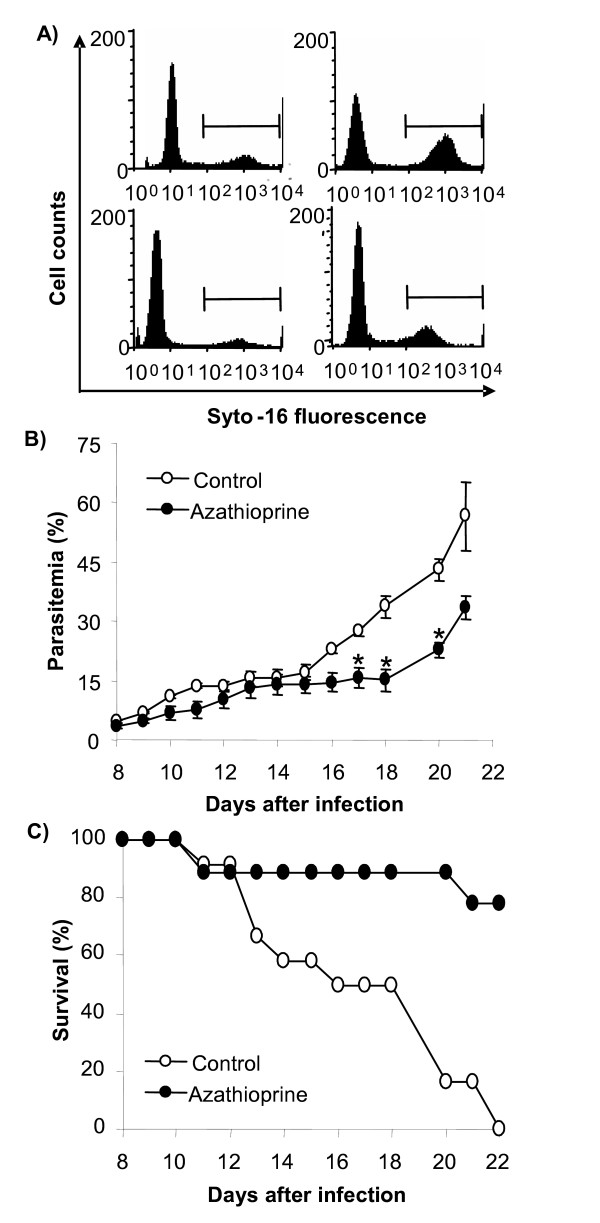
**Parasitaemia and survival of *Plasmodium berghei*-infected mice**. **A:** Original histograms of parasitaemia-dependent Syto16 fluorescence in untreated animals (upper panels) and animals treated from day 8 until day 20 with 5 mg/kg b.w. of azathioprine s.c. (lower panels) 10 (left panels) and 20 (right panels) days after infection with *P. berghei*. **B:** Arithmetic means ± SEM of parasitaemia in mice without treatment (open circles, n = 12) or with 5 mg/kg b.w. of azathioprine s.c. (closed circles, n = 9) as a function of days after infection with *P. berghei*. * indicates significant difference (p ≤ 0.05; t-test) from the untreated animals. **C**: Survival of mice without treatment (open circles) or with 5 mg/kg b.w. of azathioprine s.c. (closed squares) as a function of days after infection with *P. berghei*.

In a last series of experiments, mice were infected with *P. berghei *to determine the *in vivo *efficacy of azathioprine treatment. The administration of azathioprine (daily injections of 5 mg/kg b.w. azathioprine subcutaneously) was initiated 8 days after infection. At this time, parasitaemia was less than 5% (Figure 4B). The percentage of infected erythrocytes gradually increased in both, treated and untreated mice. The percentage of parasitized erythrocytes was lower in azathioprine-treated animals than in animals without azathioprine treatment, an effect reaching statistical significance between day 17 and day 20 of infection (Figures 4A and 4B). Accordingly, azathioprine treatment at least transiently decreased parasitaemia (Figure 4A, right panels and Figure 4B).

Azathioprine treatment further affected the survival of *P. berghei*-infected mice. As illustrated in Figure 4C, all untreated animals died within 22 days after the infection. In contrast, 77% of the azathioprine-treated animals survived the infection for more than 22 days.

## Discussion

The present study unravels a novel effect of azathioprine, i.e. the favorable influence on the course of malaria. Most importantly, azathioprine treatment significantly enhances the percentage of surviving animals after infection with *P. berghei*. As shown previously, without treatment, the infection of mice with *P. berghei *is followed by an invariably lethal course of malaria within 22 days [46]. In contrast, most of the mice treated with azathioprine survived the infection for 22 days.

Several mechanisms may contribute to the efficacy of azathioprine. In theory, the effect of azathioprine could have been due to its immune-suppressing potency [35-38]. However, it is not likely that immunosuppression achieves both, a significant reduction of parasitaemia and a milder course of the disease.

Azathioprine could further affect parasitaemia and host survival by directly affecting the survival and replication of the pathogen or its ability to evade parasitized erythrocytes and to invade noninfected erythrocytes. Indeed, higher concentrations of azathioprine decreased *in vitro *parasitaemia and DNA/RNA content of parasitized erythrocytes.

The effect of azathioprine could further be secondary to its ability to stimulate suicidal death of erythrocytes [39], an effect, which could contribute to or even account for the blunted parasitaemia and the survival of the infected mice. The drug could be effective by accelerated clearance of infected erythrocytes due to eryptosis. Moreover, the enhanced eryptosis may promote the release of pro-inflammatory cytokines from activated macrophages, thereby resulting in the activation of the hormonal stress response [48].

Phosphatidylserine-exposing erythrocytes are engulfed by macrophages [21,22] and thus cleared from circulating blood [23]. A wide variety of further endogenous mediators and xenobiotics trigger eryptosis, including haemolysin Kanagawa [49], listeriolysin [50], PGE_2 _[51], Bay-5884 [52], platelet activating factor [53], chlorpromazine [54], anandamide [55], methylglyoxal [56], paclitaxel [57], curcumin [58] amyloid peptides [59], valinomycin [60], aluminium [61], lead [62], mercury [63] and copper ions [64]. Moreover, eryptosis is enhanced in a variety of clinical conditions including iron deficiency [23], sickle-cell anaemia [65,66], beta-thalassaemia [7], glucose-6-phosphate dehydrogenase (G6PD)-deficiency [7], phosphate depletion [67], Haemolytic Uremic Syndrome [68], sepsis [69], malaria [25] and Wilson disease [64]. Several of those diseases and xenobiotics have already been shown to favorably influence the course of malaria, including sickle-cell trait, beta-thalassaemia-trait, homozygous Hb-C and G6PD-deficiency [7,26-30], iron deficiency [1], lead [2], chlorpromazine [31] and cyclosporine [32]. Azathioprine may be a particularly attractive substance for the treatment of malaria because it is clinically widely used and thus ample knowledge has been accumulated about its side effects. Nevertheless, further eryptosis-inducing substances may be shown in near future to be effective as antimalarial drugs.

## Conclusion

In conclusion, azathioprine accelerates eryptosis of *Plasmodium*-infected erythrocytes The effect contributes to or even accounts for the favourable effect of azathioprine on parasitaemia and survival of the host during malaria.

## Abbreviations

ANOVA: Analysis of variance; APC: Allophycocyanin; DNA: Desoxyribonucleic acid; FACS: Fluorescence activated cell sorting; FL: fluorescence channel; G6PD: Glucose 6 phosphate dehydrogenase; HEPES: N-2-hydroxyethylpiperazine-N-2-ethanesulfonic acid; Hb-C: Haemoglobin C; *P*: *Plasmodium*; RNA: Ribonucleic acid.

## Competing interests

The authors declare that they have no competing interests.

## Authors' contributions

DB performed the *in vitro *experiments, SK performed the *in vivo *experiments, CG performed FACS analysis, MF participated in the design of the study and the FACS analysis, evaluated the results and made the illustrations. SMH participated and supervised the *in vitro *and *in vivo *experiments, FL designed the study and drafted the manuscript. All authors read and approved the final manuscript.
